# Total flavonoids from *Semen Cuscutae* target MMP9 and promote invasion of EVT cells via Notch/AKT/MAPK signaling pathways

**DOI:** 10.1038/s41598-018-35732-6

**Published:** 2018-11-26

**Authors:** Feixia Gao, Chun Zhou, Weiyu Qiu, Haiwang Wu, Jing Li, Jinting Peng, Min Qiu, Chun Liang, Jie Gao, Songping Luo

**Affiliations:** 1 Shanghai Institute of Biological Products, Shanghai, 200050 China; 2 Guangzhou University of Chinese Medicine, Guangzhou, 510405 China; 3 Medical Immunopharmacology Research Center, School of Pharmaceutical Sciences, Southern Medical University, Guangzhou, China; 4 Department of Gynecology, No. 1 Affiliated Hospital to Guangzhou University of Chinese Medicine, Guangzhou, China; 5 Division of Life Science, Center for Cancer Research and State Key Lab for Molecular Neural Science, Hong Kong University of Science and Technology, Hong Kong, China

## Abstract

Miscarriage is a common condition during pregnancy and its mechanisms remain largely unknown. Extravillous trophoblast (EVT) cell invasion is required to maintain normal pregnancy and its malfunction has been proposed as a major cause for miscarriage. Homeostasis of matrix metalloproteinase 9 (MMP9) is a key to regulate EVT cell invasion. Total flavonoids from *Semen Cuscutae* (TFSC) have been applied clinically used for preventing or treating miscarriage in the past. Given its potential clinical benefit on preventing miscarriage, this study aims at examining the therapeutic effect of TFSC in the prevention of premature birth by upregulating MMP9 and promote EVT cell invasion. HTR-8 cells migration and invasion functions were analyzed using wound healing and transwell assays. The regulatory effect of TFSC on MMP9 expression and relevant signaling pathways were analyzed by Western Blot. The results show compared to control group, TFSC significantly promoted the migration of EVT cells in a dose and time-dependent manner. The migration and invasion of EVT cells were maximized at the highest dosage of 5 μg/ml of TFSC. The expression of MMP9 in EVT cells was significantly increased after TFSC treatment. Furthermore, cells treated with TFSC significantly upregulated protein expressions in Notch, AKT and p38/MAPK signaling pathways. We believe TFSC can promote the migration and invasion of EVT cells by increasing MMP9 expression, and prevent miscarriage by activating Notch, AKT, and MAPK signaling pathways.

## Introduction

During embryogenesis, the synergistic functions of proliferation, migration and invasion of EVT cells play essential role to ensure nutrient intake of embryos and normal pregnancy^1^. The dysfunction of EVT cells may lead to a range of abnormalities including fetal anomalies, growth retardation, miscarriages and stillbirths. In particular, excessive invasion ability may lead to life threatening conditions in the mother like placenta accreta, choriocarcinoma or hemorrhage. In contrast, insufficient invasion ability may cause some common clinical obstetrical and gynecological conditions such as miscarriage, preeclampsia and growth retardation of the fetuses^2,3^. While the invasion of EVT cells shares similarities to tumorigenesis, its process is highly spatial and temporal regulated^4^.

The invasion of EVT cells depends on the dissolution and reconstruction of extracellular matrix of decidual cells mediated by MMPs, indicating an essential role of MMPs in promoting the invasion of EVT cells^5^. MMP9, a family member of MMPs, plays an irreplaceable role in fetal nidation and placental implantation during early pregnancy^6^. Moreover, the normal secretion of MMP9 by EVT cells is important in maintaining its invasion ability^7^. The deficiency of MMP9 during early pregnancy impaired the abilities of differentiation and invasion of EVT cells, eventually leading to fetal abnormities, intrauterine growth restriction and even stillbirths in rats^8^. Therefore, by investigating the MMP9 expression in EVT cells, one can gain a better understanding of their invasion function during pregnancy.

*Semen Cuscutae* is widely used in China, and total flavonoids from *Semen Cuscutae* (TFSC) is a main estrogenic active constituent of the plant which include hyperoside, quercetin, kaempferol and rutin, isorhamnetin^9,10^. It could improve liver and kidney function, treat impotence and seminal, improve testosterone level (AR level) and androgen receptor gene expression, reverse the kidney-yang deficiency and prevent miscarriage^9,11^. In addition, TFSC involve in hippocampal-hypothalamic-pituitary-ovarian (HPO) axis, by influence the levels of hormone and hormone receptors such as ER, LHR, which might be the target molecules of TFSC. This may be the reason that TFSC involved in HPO axis by regulating ER and LHR could treat ovarian endocrine and reproductive dysfunction and prevent miscarriage^12,13^. Previous studies have confirmed that the potency and biological effects of flavonoids were mainly contributed by TFSC. According to modern pharmacology studies, the main therapeutic effects of TFSC include improving reproductive endocrine system, protection of cardiac and cerebral vessels as well as regulation of the immune system and bone metabolism via antioxidative and antiapoptotic effect, and moreover, prevent abortion by regular the function of trophoblast and deciduas^14^. Currently, the role of TFSC on EVT cell migration and invasion has not been reported, and it is still largely unclear how TFSC influence the cellular functions of EVT cells.

Numerous reports have demonstrated that the expression of MMP9 was regulated by MAPK, PI3K/AKT and Notch signaling pathways, which are involved in the regulation of survival, proliferation and invasion abilities of cells. Some results showed that CsA promoted the invasion of EVT cells as well as increased MMP9 and MMP2 expression during early pregnancy via activating MAPK signaling pathway^15^. In addition, both NME1 suppression and CCL2 could promote the proliferation and invasion abilities of endometrial stromal cells (ESCs) by upregulating MMP9 expression via AKT and MAPK signaling pathways^16,17^. It has also been reported that PIM2 upregulated COX-2 and MMP9 expression via PI3K signaling pathway, which was in turn mediated by Notch1^18^. In spite of these evidences, studies on the relationship between MMP9 and the regulating effects of Notch, PI3K/AKT and MAPK signaling pathways in EVT cell invasion remain scarce and more in-depth analysis is needed to uncover the underlying molecular mechanism.

In order to better understand the pharmacodynamics of TFSC, in this study we investigated how TFSC regulate the migration and invasion abilities of HTR-8 EVT cells, and its role in MMP9 expression and downstream signaling cascades such as Notch, AKT and MAPK pathways involved.

## Materials and Methods

### Cells, reagents and antibodies

HTR-8 extravillous trophoblast strains (EVT cells) were purchased from Jiangsu BeNa Culture Collection, Jiangsu, China. TFSC extracts (≥99%) were purchased from Zhenwo Biomedical Technology, Anhui, China. RPMI-1640 culture medium, PBS, pancreatin, and FBS from Gibco. MTT and DMSO were from Sigma. Transwell chambers (pore size: 8 μm) were from Costar. BD Matrigel (354243). P-MAPK/MAPK Family Ab sample kit (9910 S), Notch isoform Ab sample kit (3640S), and Phospho-Akt/AKT pathway antibody sample kit (9916S) were from CST. Anti-MMP9 antibodies (1957970) were obtained from Millipore. Anti-Tubulin antibodies (AT819-1) and the kits for protein extraction, quantification and gel preparation (P0012A) were from Beyotime, Jiangsu. ECL substrate was from Bio-Rad. Hyperin, Rutin, Quercetin, Quercitrin and Isorhamnetin were from Chinese Academy of food and Drug Administration

### HTR-8 cell culture

HTR-8 cells were cultured in RPMI-1640 media supplemented with 10% fetal bovine serum (FBS) and 1% antibiotics (penicillin/streptomycin) at 37°C, 5% CO_2_. Cells were passaged with treatment of 0.25% trypsin and resuspended in 10% RPMI-1640 medium when most of the cells detached.

### Cell viability assay

Cell viability was analyzed by thiazolyl blue tetrazolium bromide (MTT) assay. HTR-8 cell lines were cultured in 96-well plates at the density of 1.5 × 10^4^ cells/ml, 100 μl for each well at 37°C having 5% CO_2_. After 24 h, cells were treated with TFSC at different dosages (0.1, 0.5, 1, 5, 10 μg/ml) for 48 h. 0.1% dimethyl sulfoxide (DMSO) was used as the negative control. A total of five replicates were performed for each treatment group. Then, 10 μl of 5 mg/ml MTT were added to each well, followed by 3 h incubation. The supernatant was discarded and the crystals were dissolved using 100 μl of DMSO. The plates were place on a shaker for 10 minutes at room temperature, and the optical density (OD) of each well at 490 nm was measured using an enzyme immunoassay analyzer (Thermo Scientific).

### Wound healing assay

HTR-8 cell lines were cultured in 6-well plate at a density of 5 × 10^4^ cells/ml, 2 ml for each well. After 24 h, a straight line was scratched with a sterile pipette tip and the cells originally in the area of the line were removed. Then the remaining cells were treated with TFSC with different dosages (0.5, 1 or 5 μg/ml) for another 24 h. 0.1% DMSO was used as the negative control. Images were obtained using microscope at 0 h, 6 h, 12 h, and 24 h. The extent of migration of the cells to the middle of the scratch was measured under light microscope with green colour to image the cells under 40 x magnifications, without any green stain, and a mean value of migrating distance was calculated.

### Transwell invasion assay

50 μl of matrigel was diluted with 200 ul of serum-free RPMI 1640 medium and added to the 24-pore transwell upper chamber for 1 h incubation at 37°C. 200 μl of EVT cells were cultured in the upper chamber with a density of 5 × 10^4^ cells/ml, and 500 μl of RPMI-1640 medium having 10% FBS was placed in the lower chamber. Different concentrations of TFSC (0.5, 1 or 5 μg/ml) were added to the upper chamber, 0.1% DMSO was used as the negative control. The cells were then incubated for 48 h at 37°C, 5% CO_2_. After rinsed three times with PBS, the cells on the upper side of the filter membrane were gently removed with cotton swabs. The cells on the lower side of the membrane were fixed with paraformaldehyde for 30 minutes, followed by 20 minutes of crystal violet staining. The membrane was then removed and observed under microscope. For each well, five views were randomly selected in 400x microscopic fields. The invasion index treated by different TFSC concentrations were calculated according to the following formula: [invasion index = number of cells in the experiment group/number of cells in the control group]. The experiment was repeated three times and the mean values were taken for statistical analysis.

### Quantitative RT-PCR

Total RNA was extracted using Magzol and reverse-transcribed (RT) into cDNA (Thermo Scientific) according to the manufacturer’s instructions. Quantitative real time PCR (qPCR) was performed using SYBR Green II Master Mix (Thermo Scientific) in iCycler iQ thermocycler (Bio-Rad). The thermal cycling program was as follows: initial denaturation at 95°C for 30 s, and 40 cycles at 95°C for 5 s and 60°C for 20 s. All the primers used for qPCR analysis were purchased from Invitrogen. The specificity of each primer pair was confirmed by melting curve analysis and agarose-gel electrophoresis. β-actin was used as an internal control for normalization. Sequences of primers (5′ to 3′) are as follows: for β-actin, forward: GCCGCCAGCTCACCAT, reverse: AATCCTTCTGACCCATGCCC, MMP9, forward: GCTACGTGACCTATGACATCCT; reverse: TCCTCCAGAACAGAATACCAGTT.

### Western blot analysis

Cells were treated with TFSC in different dosages (0.1, 0.5, 1, 5, 10 μg/ml) for 12 h and then whole cell proteins were extracted using a pre-cooled RIPA buffer with PMSF. Equivalent amounts of proteins (20 µg) from the soluble fractions of the lysates were separated by 10% SDS–PAGE, and transferred to NC membrane (Merk, Millipore). After blocking with 5% nonfat milk in TBS containing 0.1% Tween 20 for 1 h at room temperature (RT), the membranes were incubated with primary antibodies (1:500-1:1000) at 4 °C overnight, and then probed with horseradish peroxidase-conjugated secondary antibodies for 1 h at RT. Immunoreactive bands were detected by enhanced chemiluminescence and visualized with the GelDoc (Bio Rad). The intensities of the blots were quantified by densitometry using ImageJ software according to manufacturer’s instructions.

### HPLC analysis

Reference solution preparation was performed by dissolving a standard amount of TFSC in methanol and test solution preparation was prepared by placing a standard amount of TFSC in a 50 ml round-bottom flask with methanol poured in. After ultrasonic treatment for 30 minutes, the solution was left to cool and topped up with another 50 ml of methanol. Then the supernatant was collected and filtered it a membrane (pore size 0.45μm). After filtration, the supernatants were injected into the ultra-performance liquid chromatography (Waters). The conditions of chromatographic were as follows: chromatographic column: Ecosil C18 columns (4.6*250 mm), mobile phase, methanol-0.4% phosphoric acid water, column temperature 30°C, injection volume 10 μl, detection wavelength 360 nm, gradient elution, 0 min, methanol-0.4% phosphoric acid water (25:75), 60 min, methanol-0.4% phosphoric acid water (40:60), 80 min, methanol-0.4% phosphoric acid water (60:40).

### Statistical analysis

All the data were processed on the SPSS19 software. The ANOVA method was used for comparisons among groups and t-tests were used on continuous variables to verify the comparison between groups. GraphPad Prism 5 was used for drawing charts. P < 0.05 indicates statistical significance. All the experiments were performed in triplicates.

## Results

### TFSC purity was determined by High-performance liquid chromatography (HPLC) analysis

**Figure 1:**
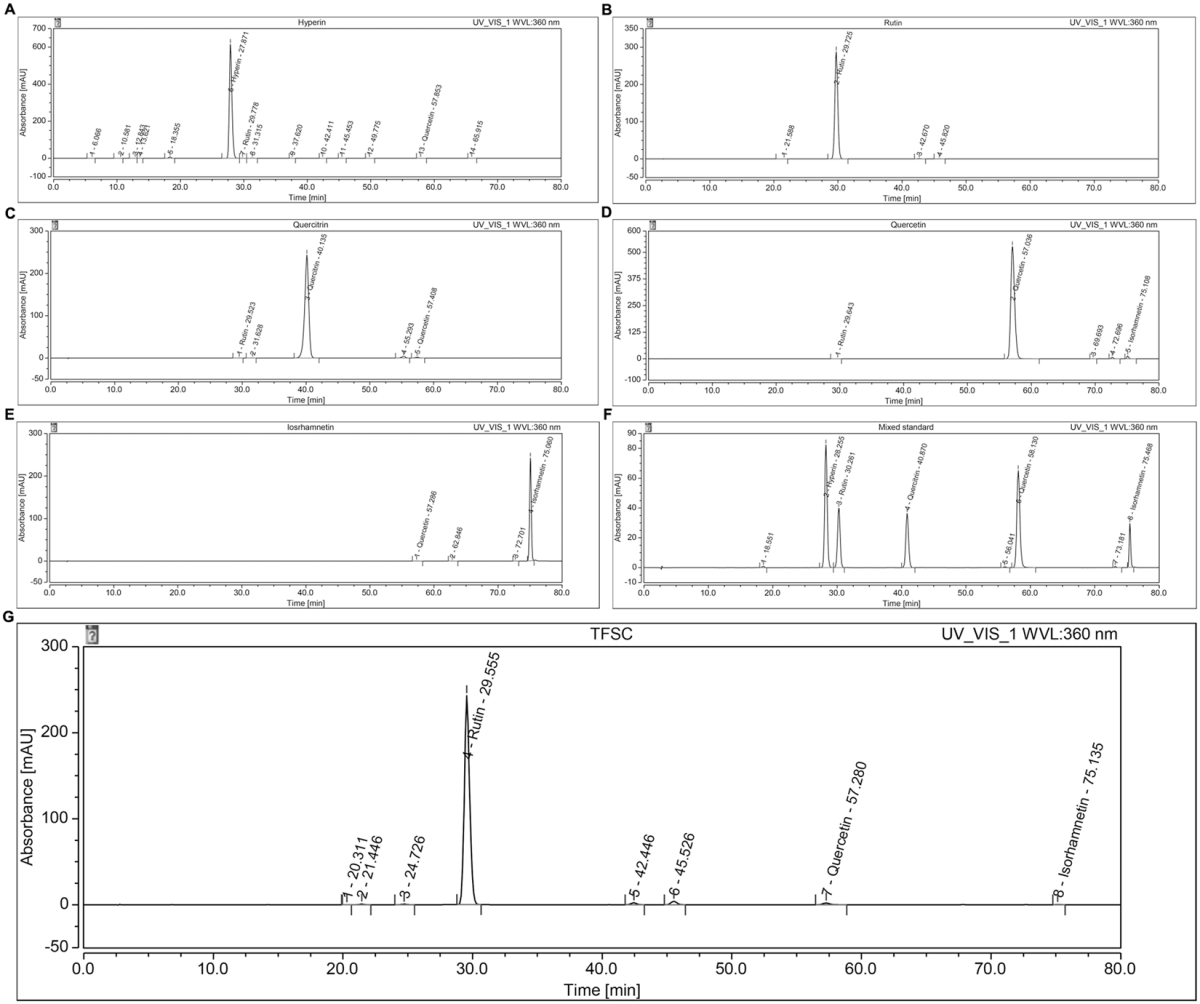
Identification of the components and respective percentage of TFSC. TFSC components evaluated (**A**) Hyperin, (**B**) Rutin, (**C**) Quercitrin, (**D**) Quercetin, (**E**) Iosrhamnetin, (**F**,**G**) Mixed standard and TFSC. (**G**)TFSC is clearly separated from rutin (Peak 4) 84.75%, quercetin (Peak 7) 7.91%, isorhamnetin (Peak 8) 6.73%, little hyperin and quercitrin. HPLC Conditions: chromatographic column: Ecosil C18 columns (4.6*250 mm), mobile phase, methanol-0.4% phosphoric acid water; column temperature, 30°C; injection volume, 10 μl, detection wavelength 360 nm, gradient elution, 0 min, methanol-0.4% phosphoric acid water (25:75), 60 min, methanol-0.4% phosphoric acid water (40:60), 80 min, methanol-0.4% phosphoric acid water (60:40).

In this study, TFSC was quantitated by HPLC. Results showed that the TFSC density averaged at 99.39% and the substances included rutin (Peak 4) 84.75%, quercetin (Peak 7) 7.91%, isorhamnetin (Peak 8) 6.73%, little hyperin and quercitrin. (Fig. 1).

### TFSC had no toxicity on EVT cells

**Figure 2:**
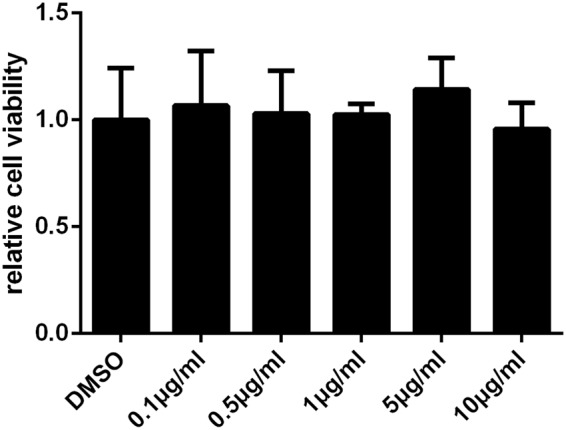
Assessment of cell viability of EVT cells treated with TFSC by MTT assay. The cell viability showed no decrease after treated with TFSC in the concentrations of 0.1, 0.5, 1, 5, 10 μg/ml. Results shown are representative of three different experiments. All values were expressed as mean ± SEM.

To determine the potential toxicity of TFSC on EVT cells, MTT assay was employed to assess cell viability 48 hours after treatment with different dosage of TFSC. The results showed that the proliferation rate of EVT cells were similar in all treatment groups and TFSC did not affect the viability of EVT cells indicating that TFSC had no significant toxicity effect on EVT cells (Fig. 2).

### TFSC promote EVT cell migration and invasion

**Figure 3:**
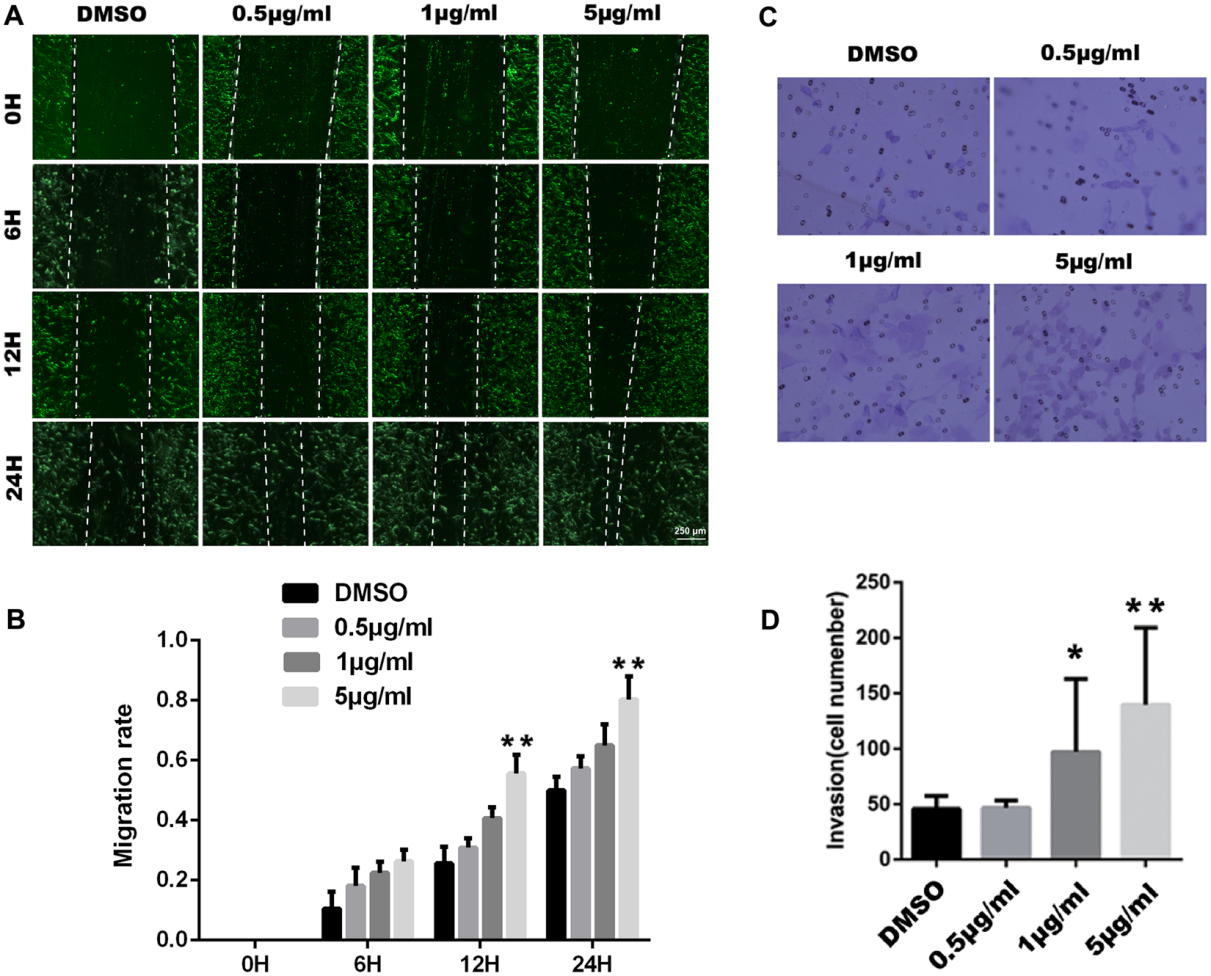
Wound healing assay and transwell assay of EVT cells. (**A**) Representative images of the wound-healing assay indicating increased migratory abilities of EVT cells with TFSC of 0.5, 1, 5 μg/ml, and at different time points; magnification: 40×, with green light microscope and without any green stain. (**B**) Quantitation of the wound healing assay results from TFSC; (**C**) Representative images of transwell invasion assays showing increased invasive abilities of EVT cells with different dosages of TFSC for 48 h; magnification: 400×. (**D**) Quantitation of the transwell invasion assay results. Data shown are the mean ± SEM of three independent experiments. *p < 0.05, **p < 0.01, ***p < 0.001, vs. Control (DMSO).

To further determine the role of TFSC in EVT cell migration and invasion (Fig. 3), EVT cells were treated with 0.5 μg/ml, 1 μg/ml, or 5 μg/ml of TFSC for 0, 6, 12, and 24 h. The wound healing assay results showed that TFSC could significantly improve the migration ability of EVT cells, in a dose- and time-dependent manner, especially in 1 μg/ml and 5 μg/ml groups. The transwell results also suggested that after treated with different doses of TFSC for 48 h, the invasion ability of EVT cells was significantly enhanced in the treatment group of 1 μg/ml and 5 μg/ml compared with control.

### TFSC enhance the MMP9 expression of EVT cells

**Figure 4:**
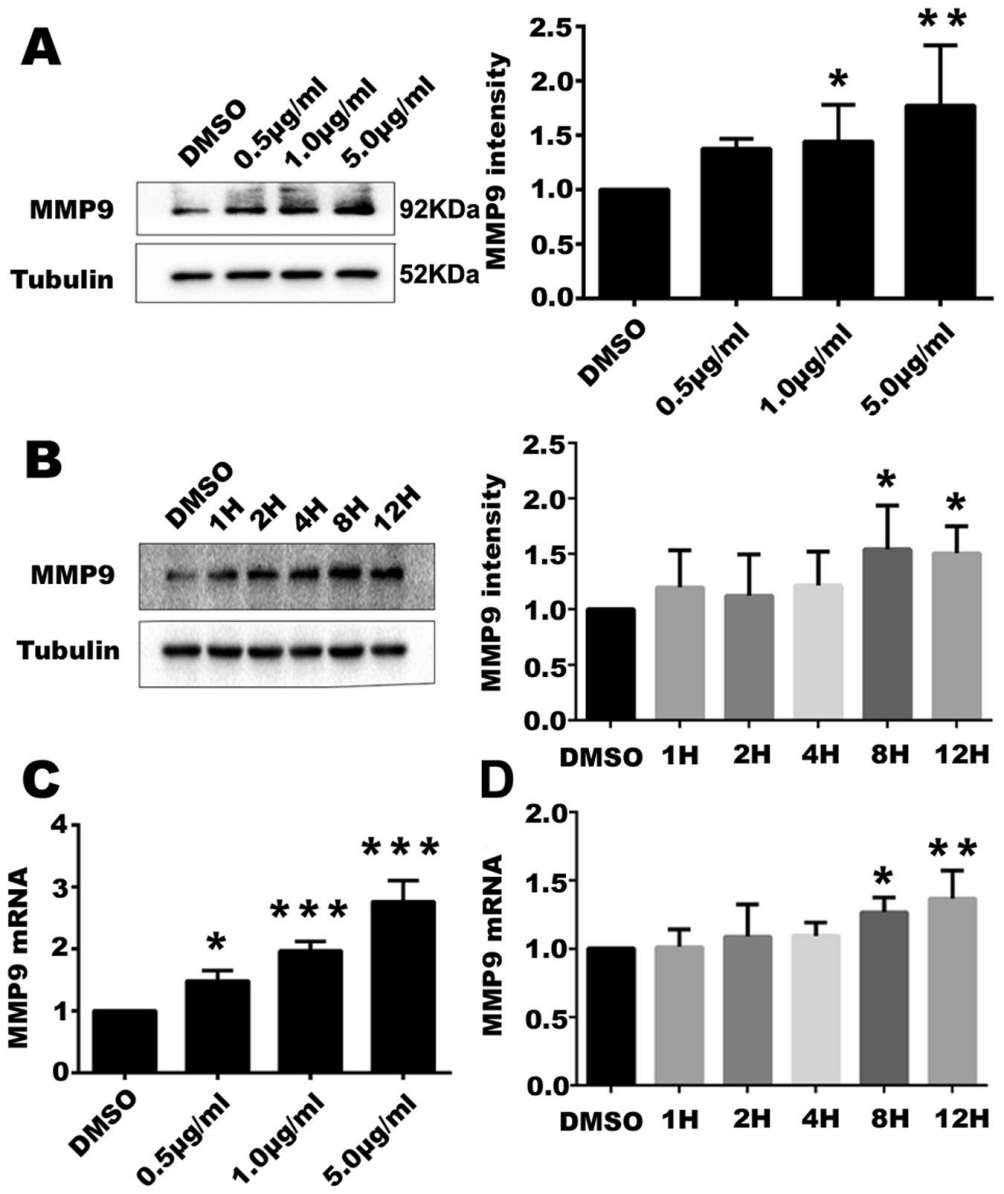
TFSC up-regulate MMP9 expression in EVT cells. (**A**,**B**) Protein expressions of MMP9 were confirmed by Western blotting with different dosages of TFSC and treatments at different time points; (**C**,**D**) MMP9 mRNA level in EVT cells were confirmed by RT-qPCR with different dosages of TFSC and treatments at different time points. Tubulin was used as an internal reference. *p < 0.05, **p < 0.01, ***p < 0.001, vs. Control (DMSO). The western blots were derived under the same experimental conditions from the same cell lysates; Data is representative of 3 dogs; each experiment is run in triplicate. Bars represent mean ± SEM.

To investigate the underlying molecular mechanism of TFSC, we examined the gene and protein expression level by PCR and Western blot in EVT cells. After treatment with TFSC for 12 h, the MMP9 protein expression levels were up-regulated significantly compared with control (DMSO) group, in a dose and time dependent manner, especially in 1 μg/ml and 5 μg/ml groups (Fig. 4A). To further determine the effects of TFSC on MMP9 expression at different time points after treatment, EVT cells were incubated with 1 μg/ml TFSC (medium dose) for 1 h, 2 h, 4 h, 8 h and 12 h. The results showed that in 8 h and 12 h groups, the MMP9 protein levels were significantly increased compared to the control group (Fig. 4B). MMP9 mRNA expression was upregulated significantly by TFSC and the expression level was most elevated at 8 h and 12 h after treatment with 1 μg/ml TFSC (Fig. 4C,D). These results indicate that TFSC can promote the expression of MMP9 in EVT cells.

### TFSC promote EVT cells invasion via Notch/AKT/MAPK signaling pathway by targeting MMP9

To further investigate the pharmacodynamics of how TFSC promoted EVT cell migration and invasion via promoting the MMP9 level, we further evaluated the Notch, ATK, and MAPK signaling pathways by Western Blot. EVT cells were treated with 0.5 μg/ml, 1 μg/ml or 5 μg/ml of TFSC for 12 h, or with 1 μg/ml for different time points. Whole cell proteins were extracted after the treatment and proceed for Western Blot.

**Figure 5:**
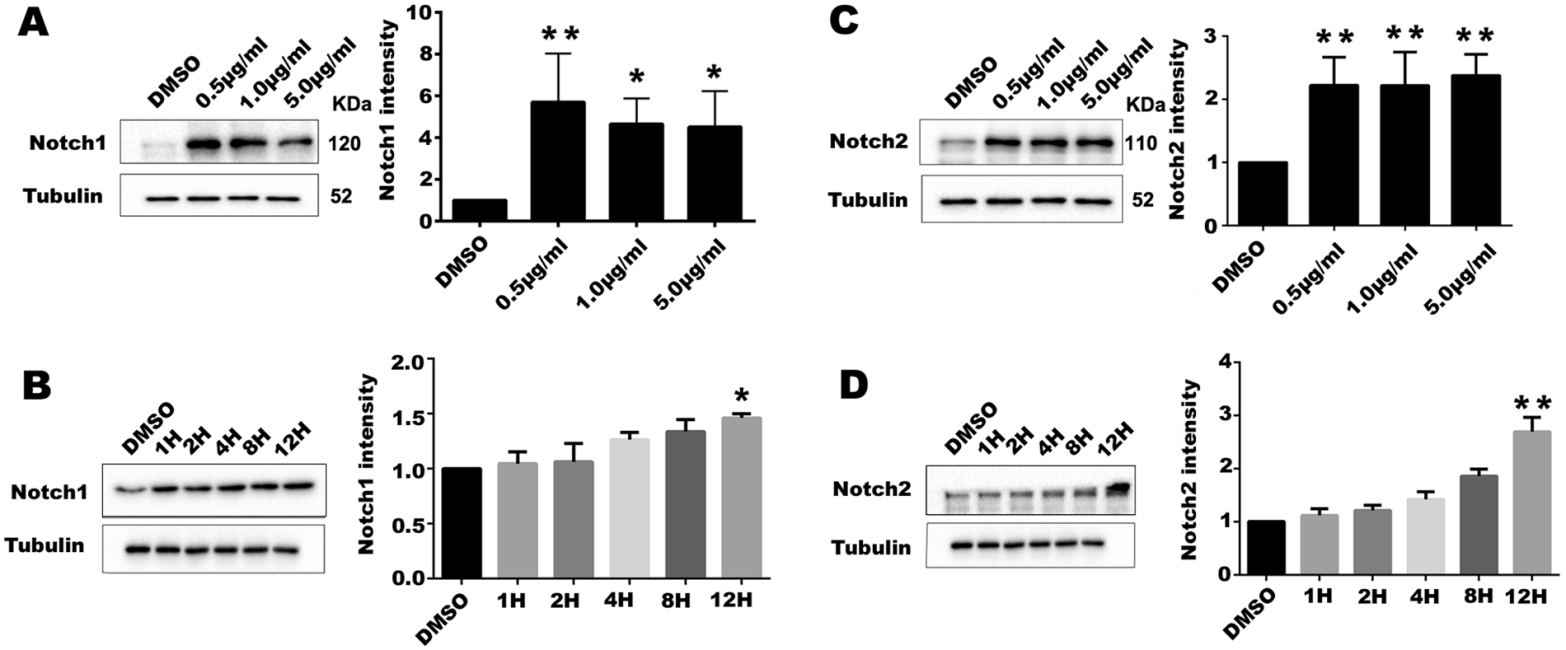
TFSC up-regulate Notch1 and Notch2 expression in EVT cells. (**A**) Western blotting analyses of Notch1 after treatment of different dosages of TFSC or at different time points (**B**); (**C**) Western blotting analyses of Notch2 after treatment of different dosages of TFSC or at different time points (**D**). Tubulin was used for normalization of protein loading. *p < 0.05, **p < 0.01, vs. Control (DMSO). Data shown are the mean ± SEM of three independent experiments.

The results showed that treatment of TFSC increased the Notch protein level in EVT cells. Compared to DMSO, the Notch1 protein level was significantly upregulated after treatment with 0.5 μg/ml, 1 μg/ml, and 5 μg/ml TFSC (Fig. 5A). Notch1 protein was marginally upregulated at 1 μg/ml of TFSC at different time points in 12 h groups (Fig. 5B). Expression of Notch 2 proteins were increased at 0.5 μg/ml, 1 μg/ml and 5 μg/ml of TFSC significantly compared to the control group with the greatest magnitude among all treatment groups, and the expression level was most elevated at 12 h after treatment with 1 μg/ml TFSC (Fig. 5C,D).

**Figure 6:**
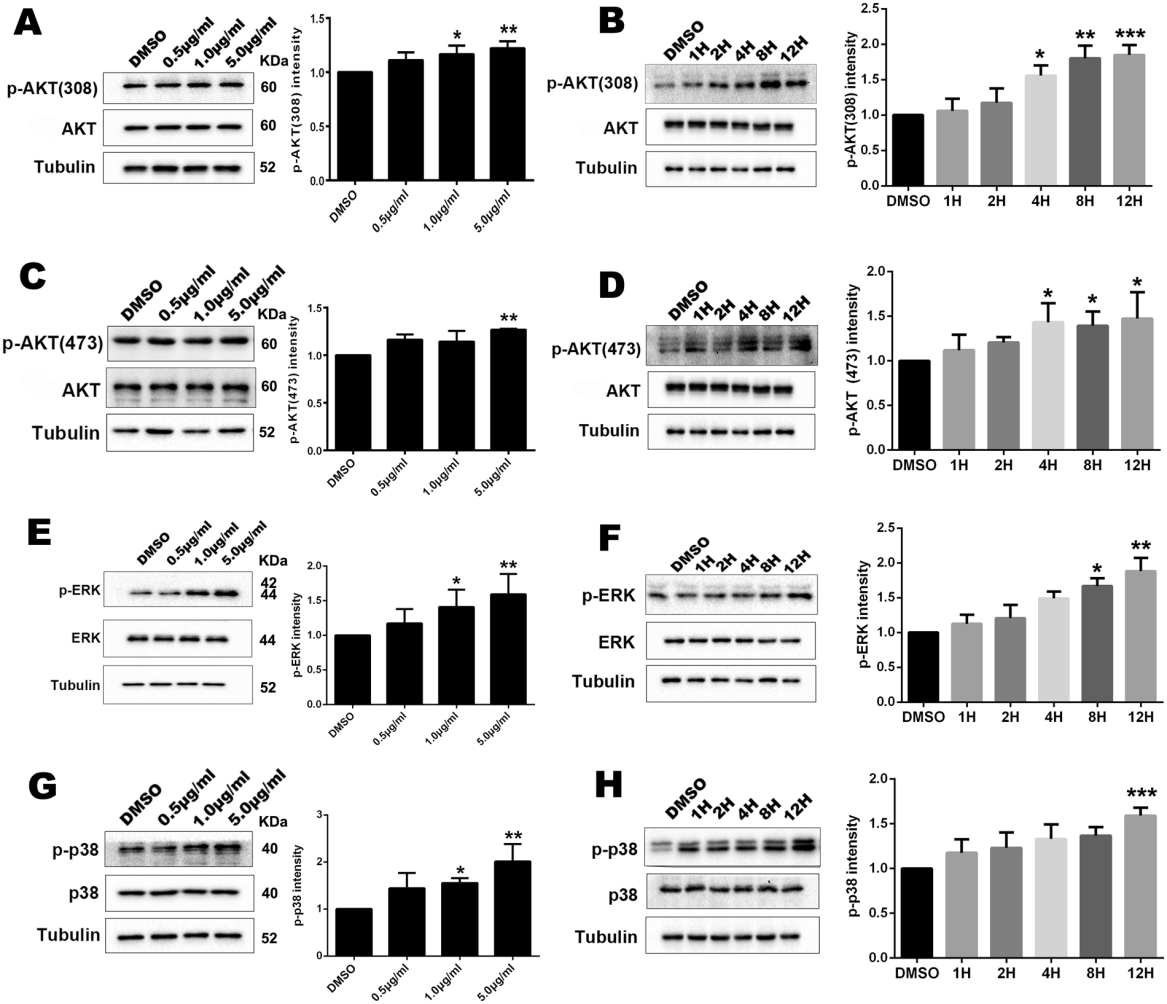
TFSC activate the AKT and MAPK signaling pathways in EVT cells. (**A**) Western blotting analyses of p-AKT(308) levels increased by different dosages of TFSC at 12 h of exposure and by 1 μg/ml of TFSC at different time points (**B**); (**C**) Protein levels of p-AKT(473) levels increased by different dosages of TFSC at 12 h of exposure and by 1 μg/ml of TFSC at different time points (**D**); total AKT unchanged (**A**–**D**); (**E**)Western blotting analyses of p-ERK levels increased by different dosages of TFSC at 12 h of exposure and by 1 μg/ml of TFSC at different time points (**F**), total ERK unchanged (**E**,**F**); (**G**) Protein levels of p-p38 levels increased by different dosages of TFSC at 12 h of exposure and by 1 μg/ml of TFSC at different time points (**H**), total p38 unchanged (**G,H**); Tubulin was used as an internal reference. *p < 0.05, **p < 0.01, ***p < 0.001, vs. Control (DMSO). Data shown are the mean ± SEM of three independent experiments.

Next, we examined the effect of TFSC on activation of AKT pathway by assessing the phosphorylation level. Compared to DMSO, 1 μg/ml and 5 μg/ml TFSC treatment increased the p-AKT (308) level in EVT cells but not the total AKT level, especially, p-AKT (308) protein was marginally upregulated at 1 μg/ml of TFSC in a time-dependent manner (Fig. 6A,B). Moreover, for p-AKT (473), 5 μg/ml of TFSC increased the phosphorylation level of AKT (473) significantly but the total protein level remained unchange, and the expression level was most elevated at 4 h,8 h and 12 h after treatment with 1 μg/ml TFSC (Fig. 6C,D).

Similar upregulation in phosphorylated proteins by TFSC were observed in ERK as well. Compared to DMSO, 5 μg/ml of TFSC significantly increased p-ERK but not the total protein level (Fig. 6E). After treatment with 1 μg/ml of TFSC, the phosphorylation levels of ERK were significantly upregulated at all time points in a time-dependent manner (8 h and 12 h) (Fig. 6F). The phosphorylation level of another key molecule of MAPK pathway, p38, was also increased substantially 12 h of treatment with 1 μg/ml and 5 μg/ml of TFSC (Fig. 6G). However, the change of p-p38 level was slightly increased with 1 ug/ml of TFSC at 12 h (Fig. 6H).

Thus, these results indicate that TFSC could promote the expression of MMP9 in EVT cells via cascade reactions of Notch, ATK, and MAPK signaling pathways, thereby improving the migration and invasion abilities of EVT cells.

## Discussion

The invasion of EVT cells is similar to tumorigenesis and plays an important role in decidual and placental implanting as well as normal development of fetuses^19^. However, the specific mechanism of invasion is still unclear and investigating the invasion of EVT cells is clinically significant for understanding the pathologies of preeclampsia and miscarriage. It has been confirmed that *Semen Cuscutae* which is widely used in China for thousand years played an important role in preventing miscarriage and its main active constituents, TFSC, are crucial for this protective effect, and previous study has reported that TFSC could involve in HPO axis by regulating ER and LHR to treat ovarian endocrine and reproductive dysfunction, regular the proliferation and apoptosis of the trophablast and deciduas, furthermore, effect on endocrinological and immunological network to prevent abortion^11,12,14^. However, the mechanism of how treatment with TFSC prevents miscarriage is yet to be uncovered. In spite of MMP9’s significant role in cell invasion ability^6^, few studies have reported on the role and mechanisms of TFSC on MMP9 expression.

In this paper, we found that TFSC mainly contained rutin, quercetin, and a small amount of isorhamnetin, corresponding to previous studies where *Semen Cuscutae* extract is mainly consisted of rutin, isorhamnetin, a small amount of hyperin and kaempferol^10,20^. Based on the HPLC result, rutin may be the key therapeutic reagent present in TFSC.

Here, we demonstrated that TFSC could promote the migration and invasion abilities of EVT cells in a dosage-dependent manner, which correspond to the previous studies on TFSC promoting proliferation of EVT cells. The results also showed that MMP9 contributed to the migration and invasion of EVT cells, which might be regulated by the expression and activation of MMP9 and their inhibitor TIMP. A decrease in the level of MMP9 expression might inhibit the invasion of EVT cells, and thus cause preeclampsia. Studies by Jia and colleagues indicated that CDX2 could promote the invasion ability of EVT cells via regulating the MMP9 expression^21^. In the current study we observed that after treatment with TFSC, the MMP9 expression of EVT cells were significantly upregulated, with significant concurrent enhancement in migration and invasion abilities. In addition, the effects were maximized at highest dosage of 5 μg/ml. These results indicate that TFSC might promote the invasion ability by reinforcing the expression of MMP9.

In order to investigate the mechanism of TFSC, we further analyzed the Notch/AKT/MAPK signaling pathways. Previous studies have reported that MAPK signaling pathway is involved in the proliferation, migration, invasion, differentiation, and apoptosis of EVT cells^22,23^. Under most circumstances, their migration and invasion abilities were further enhanced by the activation of MAPK signaling pathway. For example, EGF could promote secretion of MMP9 and TIMP1 from EVT cells through simultaneous activation of PI3K and MAPK signaling pathways^24^. Similarly TNF-α and LAMA4 could also induce MMP9 expression in EVT cells and promote its invasion ability via MAPK signaling pathway^25,26^. Our study demonstrated that the increase in MMP9 expression of EVT cells might be regulated through the activation of MAPK signaling pathway when treated with TFSC for 12 h. Furthermore, the phosphorylation of ERK, p38 and AKT also increased with the total protein expression unchanged. The phosphorylation was shown to be most evident at later time point like 12 h. Previous study has reported that activating PI3K/AKT signaling pathways could promote the migration and invasion abilities of EVT cells, during which AKT1 and AKT3 participated in the migration process while AKT2 participate in the metabolic process or the survival of cells^27^. In our studies, TFSC increased the phosphorylation of AKT at 473 and 308. It has been reported that Notch1 regulated MMP14 expression by activating the PI3K/AKT signaling pathway. Moreover the effect of MMPs on the invasion ability of tumor cells also depends on Notch signaling pathway^28^. In another study by Palomero, crosstalk of PI3K/AKT and Notch1 pathways were also observed in a variety of cell types^29^. In particular, Notch signaling pathway plays a crucial role in the maternal-fetal interface in order to maintain the normal placentation, implantation, decidualization and receptivity of maternal-fetal interface^30,31^. It is also involved in the regulation of the invasion and differentiation of EVT cells^32,33^. We report here that TFSC could simultaneously increase the expression of Notch1 and Notch2 levels significantly, which might be caused by the participation of Notch signaling pathway in the up-regulation of MMP9 expression of EVT cells. CCN3 could also regulate the proliferation and migration of JEG-3 cells via ERK1/2, AKT and Notch/p21 signaling pathways in both glycosylated and non-glycosylated forms, accompanied with an increase in MMP2 and MMP9 expression^34^, which was in accordance with our previous findings about the synergistic effects of Notch, AKT and MAPK signaling pathways. Until now, no studies have ever reported on the relationship between TFSC and Notch, AKT, MAPK signaling pathways. Our results indicate that TFSC could promote the migration and invasion abilities of EVT cells via Notch, AKT and MAPK signaling pathways by increasing the MMP9 expression. It is plausible that Notch signaling pathway might be the link between MAPK and AKT signaling pathways. Based on the findings, it can be speculated that similar negative feedback regulation during placentation may be activated in order to control the degree of invasion of EVT cells.

Previous studies also reported similar findings that Notch1 signaling pathway could promote the migration and invasion abilities of EVT cells as well as increase the secretion of MMP9. Knockdown of Notch1 by siRNA suppressed the NF-κB signaling pathway and MMP9 expression, thereby inhibiting the migration and invasion of EVT cells^35^. Hence, more experiments are needed to clarify how Notch signaling pathway mediates the activation of MAPK and AKT signaling pathways in MMP9 expression upregulation in EVT cells when treated with TFSC. In summary, in this study we report for the first time that TFSC could promote the migration and invasion abilities of EVT cells by regulating MMP9 expression mediated via Notch, AKT and MAPK signaling pathways. These findings contribute to better understanding of TFSC’S therapeutic effect and its underlying molecular mechanism of negative feedback regulation, which in turns regulates the excessive invasion of EVT cells. The findings will help to explore the pathogenesis of invasion-related diseases such as miscarriage, preeclampsia and intrauterine growth restriction in detail and also provide guidance for disease prevention.

However, our research has some limitations. In this study, only HTR-8 cell lines were used as the research object to study the effect of TFSC on the functions of EVT cells, not the original EVT cells. Therefore, TFSC might not have the same effect in the primary EVT cells. HTR8/SVneo may be different from primary trophoblasts when responding to hypoxia^36^. However, previous study reported the use of HTR-8 cell lines to study the invasion function of PAPPA2 to inhibit the HTR-8 cell lines, also found that PAPPA2 can inhibit the invasion of primary EVT cells, indicating that it is feasible to use the HTR-8 cell model as a primary EVT model^37^. Nevertheless, it is necessary that our experiments need to be extended further using first trimester placenta-derived EVTs to study the effect of TFSC on primary EVT cells, and added experiments *in vivo* if possible.

Another limitation of our study is only to explain TFSC regulation of MMP9 and invasion, while the effects of loss/gain of MMP9 itself on invasion were not assessed. However, the deficiency of MMP9 during early pregnancy impaired the abilities of differentiation and invasion of EVT cells, eventually leading to fetal abnormities, intrauterine growth restriction and even stillbirths in rats^8^. Previous study reported MMP9 plays an irreplaceable role in EVT invasion, MEHP could inhibit the invasion of HTR8/SVneo and the activity of MMP9 at the same time, and up-regulate TIMP-1 expression. It indicated that the balance between MMP9 and TIMP-1 could effect and be essential for HTR8/SVneo invasion^38^. Therefore, in this study, we hypothesized that TFSC might inhibit HTR-8 invasion by the loss function of MMP9, which needed to further explore in our experiments.

Collectively, this study demonstrate the effectiveness of TFSC on EVT cell lines via Notch/AKT/MAPK signaling pathways by targeting MMP9.

## Conclusions

In conclusion, TFSC could promote the migration and invasion abilities of EVT cells by upregulating the MMP9 expression via activating Notch, AKT and MAPK signaling pathways.
